# Preoperative AminoIndex Cancer Screening (AICS) abnormalities predict postoperative recurrence in patients undergoing curative resection for non-small cell lung cancer

**DOI:** 10.1186/s12885-020-07575-w

**Published:** 2020-11-12

**Authors:** Masahiko Higashiyama, Ryohei Miyazaki, Hiroshi Yamamoto, Takashi Anayama, Shinya Kikuchi, Kentaro Hirohashi, Jiro Okami, Tomohiro Maniwa, Toru Kimura, Kazumasa Orihashi, Fumio Imamura

**Affiliations:** 1 Department of General Thoracic Surgery, Osaka International Cancer Institute, Osaka, Japan; 2 Department of Thoracic Surgery, Higashiosaka City Medical Center, Higashiosaka, Osaka, Japan; 3 Division of Thoracic Surgery, Department of Surgery II, Kochi University School of Medicine, Kochi, Japan; 4 Research Institute for Bioscience Products & Fine Chemicals, Ajinomoto Co., Inc., Yokohama, Kanagawa, Japan; 5 Department of Medical Oncology, Osaka International Cancer Institute, Osaka, Japan

**Keywords:** AICS, AICS (lung), Lung cancer, Surgery, Recurrence, Prognosis, Non-small cell lung cancer

## Abstract

**Background:**

AminoIndex™ Cancer Screening (AICS (lung)) was developed as a screening test for lung cancer using a multivariate analysis of plasma-free amino acid (PFAA) profiles. According to the developed index composed of PFAA, the probability of lung cancer was categorized into AICS (lung) ranks A, B, and C in order of increasing risk. The aim of the present study was to investigate the relationship between the preoperative AICS (lung) rank and surgical outcomes in patients who underwent curative resection for non-small cell lung cancer (NSCLC).

**Methods:**

Preoperative blood samples were collected from 297 patients who underwent curative resection for NSCLC between 2006 and 2015. PFAA concentrations were measured. The relationship between the preoperative AICS (lung) rank and clinicopathological factors was examined. The effects of the preoperative AICS (lung) rank on postoperative outcomes were also analyzed.

**Results:**

The AICS (lung) rank was A in 93 patients (31.3%), B in 82 (27.6%), and C in 122 (41.1%). The AICS (lung) rank did not correlate with any clinicopathological factors, except for age. Based on follow-up data (median follow-up period of 6 years), postoperative recurrence was observed in 22 rank A patients (23.7%), 15 rank B (18.3%) and 49 rank C (40.2%). In the univariate analysis, preoperative AICS (lung) rank C was a worse factor of recurrence-free survival (*p* = 0.0002). The multivariate analysis identified preoperative AICS (lung) rank C (HR: 2.17, *p* = 0.0005) as a significant predictor of postoperative recurrence, particularly in patients with early-stage disease or adenocarcinoma.

**Conclusion:**

Preoperative AICS (lung) rank C is a high-risk predictor of postoperative recurrence in patients undergoing curative resection for NSCLC.

**Supplementary Information:**

The online version contains supplementary material available at 10.1186/s12885-020-07575-w.

## Background

Metabolite profiling remains a relatively underrepresented field of biomarker development for identifying and characterizing various types of cancer. Metabolic changes in patients with cancer may reflect differences in the concentration of a single metabolite or alterations in the constituents of an entire metabolic pathway [1–5]. Metabolic changes in the blood of patients with cancer are closely associated with alterations in amino acid, protein, glucoside, and fatty acid metabolism. Therefore, metabolic profiles may be used to differentiate between patients with and without cancer [1, 2, 4–6]. In particular, amino acids are one of the most suitable candidates for focused metabolomics because they are ingested or endogenously synthesized and play important physiological roles as essential metabolites and their regulators [1–6].

Based on these findings, we developed a novel cancer screening test for the early detection of various types of cancers using AminoIndex™ technology, which scores health conditions and the probability of disease by a multivariate analysis with plasma-free amino acid (PFAA) concentrations included as a variable [1, 2]. We demonstrated that the PFAA concentration ratio was altered in 7 types of cancers: gastric, lung, colorectal, prostatic, gynecological, breast, and pancreatic cancers [1, 7]. In Japan, this technology, designated the AminoIndex™ Cancer Screening (AICS) test, is widely used in health and cancer screening [1, 7]. Since the lung-specific AICS (AICS (lung)) test, one of 7 cancer screening tests, identifies lung cancer based on a specific PFAA profile composed of significantly altered plasma concentrations of 6 representative amino acids (serine, glutamine, alanine, histidine, ornithine, and lysine) [1, 8, 9], it is now used to screen for lung cancer. The probability of lung cancer is noted as AICS (lung) values of 0.0–10.0, which are categorized as ranks A (0.0–4.9), B (5.0–7.9), and C (8.0–10.0). Since higher AICS (lung) values are associated with an increased risk of lung cancer, when judged as rank C in the screening, medical work-up for lung cancer is recommended as needed [1, 7–9].

However, the AICS (lung) test has not yet been sufficiently examined in clinical practice for lung cancer. Recently, we reported that the majority of lung cancer patients with high preoperative AICS (lung) values exhibited a reduction in AICS rank after curative resection, but that the absence of postoperative reduction in AICS rank was strongly associated with tumor recurrence [10]. Therefore, the AICS (lung) rank is a marker that reflects the presence or absence of lung cancer. However, the relationship between the AICS rank and clinicopathological factors of cancer remains unclear, and, thus, its clarification may lead to the AICS (lung) test becoming a screening test and predictor of recurrence.

To investigate the clinicopathological and prognostic significance of the AICS (lung) rank among patients with lung cancer on a large scale, the relationship between the preoperative AICS (lung) rank and surgical outcomes was retrospectively investigated in 297 patients who underwent curative resection for non-small cell lung cancer (NSCLC). To the best of our knowledge, this is the first study to report the prognostic importance of the AICS rank in surgically resected patients with cancer.

## Methods

### Patient recruitment and clinicopathological factors

In total, 297 patients who underwent curative resection for NSCLC at the Osaka International Cancer Institute (Osaka, Japan) or Kochi University School of Medicine (Kochi, Japan) between November 2006 and September 2015 were enrolled. Patients were excluded if they had a history of other malignancies at the time of surgery for NSCLC. The clinicopathological characteristics of the enrolled patients are summarized in Table 1. Their mean age was 65.3 years (range, 30–86), and there were 187 males and 110 females. A total of 112 patients were non-smokers, while 185 were current or ex-smokers. Elevated preoperative serum CEA levels were observed in 72 patients. The surgical procedure selected was standard surgery (including lobectomy or more than lobectomy) for 224 patients and limited surgery (including segmentectomy or wedge resection) for 73, with all undergoing curative resection. The histological types were adenocarcinoma in 216 patients, squamous cell carcinoma in 61, and other types in 20. Regarding the pathological stage (p-stage), 163, 58, 9, 20, 30, 16, and one case were classified as stage IA, IB, IIA, IIB, IIIA, IIIB, and IV, respectively. Stage IV disease was due to a postoperative status of preceding brain metastasis. These stage classifications were based on the 7th Edition of the TNM classification [11].

**Table 1:**
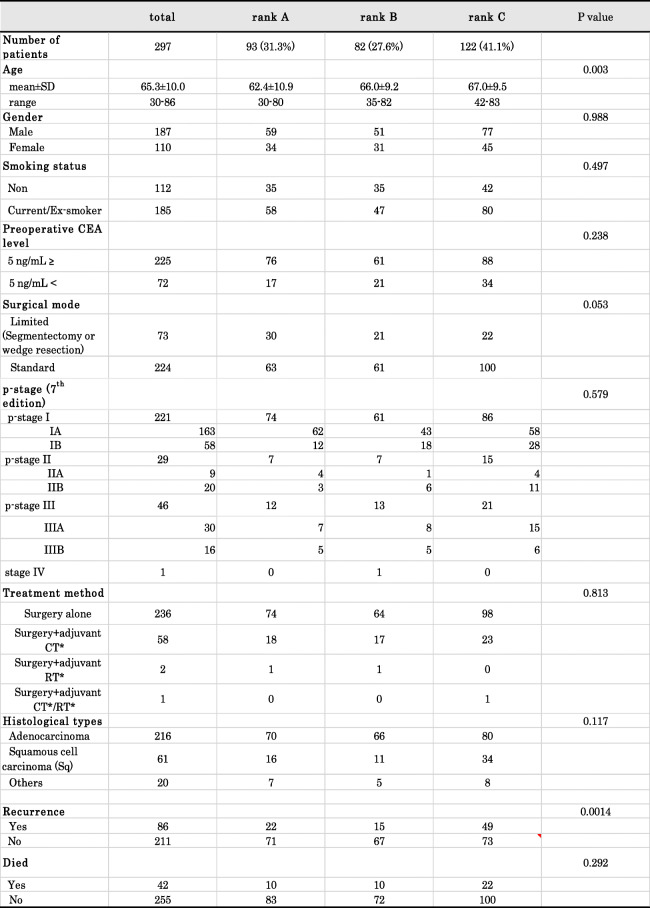
Relationship between AICS (lung) ranks and cl inicopathological factors

* CT: chemotherapy,RT: radiation therapy

Postoperative adjuvant therapy was administered to 61 patients: adjuvant chemotherapy to 58, adjuvant radiotherapy to 2, and adjuvant chemoradiation therapy to one. The regimen for adjuvant chemotherapy was as follows: oral UFT (Tegafur-Uracil) for 15 patients with stage I disease, for 2 with stage II disease, and for 1 with stage III disease. Gemcitabine (GEM) or vinorelbine (VNR) monochemotherapy was administered to one patient with stage I disease and 5 with stage II, respectively. Platinum (cisplatin or carboplatin)-based combined chemotherapy was administered to 4 patients with stage I disease, 6 with stage II disease, and 23 with stage III disease. Combined chemotherapy using GEM and VNR was administered to one patient with stage III disease. Most regimens were performed as described previously [12, 13]. Adjuvant radiotherapy was administered to two patients because of the short margin in the surgical stump of the bronchus in one and of the mediastinal field in the other. Adjuvant chemoradiation therapy was performed using the combined carboplatin-based regimen and 64 Gy on the surgical margin of the chest wall. No patients received preoperative therapy. Follow-up examinations were generally performed as follows: During the first 36 months after surgery, systemic and local screening examinations were conducted using blood tests, chest computed tomography (CT) scans were routinely obtained every 6 months, and fluoro-2-deoxyglucose positron emission tomography (FDG-PET) scans were performed where necessary. Brain CT or magnetic resonance imaging scans were also performed as required. During the first 2 years, these examinations were routinely performed and the findings obtained were carefully assessed. Thereafter, intensive examinations were performed as needed. Therefore, the median postoperative follow-up time was 6.0 years (range, 0.1 to 11.5 years) in this cohort.

The time of initial recurrence was assessed based on the onset of clinical symptoms, the detection of blood test abnormalities (e.g., serum CEA levels), or the detection of recurrent lesions on imaging, and the recurrence-free survival (RFS) period was defined as the period between surgery and the time of initial recurrence. When a tumor recurred, the initial recurrence site was evaluated and generally categorized into three patterns: local, distant, and multiple.

### Measurement of PFAA concentrations

Patients underwent preoperative blood sampling within 1 week prior to surgery. Preoperative blood samples (5.0 mL) were collected from a forearm vein on the morning after overnight fasting using tubes containing ethylenediaminetetraacetic acid, and were immediately placed on ice. Plasma was prepared by centrifugation at 3000 rpm at 4 °C for 15 min and stored at − 80 °C until analyzed. Plasma samples were deproteinized using acetonitrile at a final concentration of 80.0%. PFAA concentrations were measured using high-performance liquid chromatography/electrospray ionization tandem mass spectrometry with pre-column derivatization, which have been described previously [8, 10, 14].

### AICS (lung) values and ranks

AICS (lung) values for patients with NSCLC were classified into the following three groups: rank A, B, or C. The AICS (lung) test detects lung cancer from plasma concentrations of serine, glutamine, alanine, histidine, ornithine, and lysine. AICS (lung) values range between 0.0 and 10.0, with 5.0 producing 80.0% specificity and 8.0 producing 95.0% specificity. A high AICS (lung) value was associated with a greater probability of lung cancer. Rank A was considered to be normal (0.0–4.9), rank B was relatively high (5.0–7.9), and rank C was high (8.0–10.0). Rank C indicated a high risk of lung cancer [1, 2, 8, 9].

### Statistical analysis

RFS and overall survival (OS) curves were calculated using the Kaplan Meier method, and differences were examined by the Log-rank test. Cox’s proportional hazards regression model was used to perform a multivariate analysis of factors associated with RFS and OS. *P*-values were calculated using a one-way ANOVA for means or Fisher’s exact test for comparisons of rank. *P* < 0.05 was considered to indicate a significant difference. All statistical analyses were conducted using GraphPad Prism (software version 8; GraphPad Software Inc., San Diego, CA, USA) and R software (version 3.6.1; The R Foundation for Statistical Computing, Vienna, Austria).

### Ethical considerations

This retrospective study was performed in accordance with the Declaration of Helsinki, and the study protocol was approved by the Ethics Committees of the Osaka International Cancer Institute (Osaka, Japan) (formerly the Osaka Medical Center for Cancer and Cardiovascular Diseases) (1404015008) and the Kochi University School of Medicine (Kochi, Japan) (ERB-000486). All participants gave their written informed consent for inclusion in this study. All data were analyzed anonymously.

## Results

According to the measurement of preoperative AICS (lung) values, 93 (31.3%) patients were categorized as rank A, 82 (27.6%) as rank B, and 122 (41.1%) as rank C (Table 1).

### Each AICS (lung) rank and clinicopathological factors

The relationship between each rank and clinicopathological factors was summarized in Table 1. There were more elderly patients (*p* = 0.003) in rank C. Regarding the surgical mode, standard surgery (lobectomy or more) for lung cancer was more frequently selected for rank C (*p* = 0.053). No significant differences were observed in gender, the smoking status, preoperative CEA level, p-stage, or treatment method among patients with each rank.

### Effects of each AICS (lung) rank on tumor recurrence and OS

The median follow-up period in the present study was 6.0 years, ranging between 0.1 and 11.5 years. There were 86 (30.0%) patients with tumor recurrence, and 42 (14.1%) died (Table 1). Tumor recurrence was observed in 22 (23.7%) patients with rank A, 15 with rank B (18.3%), and 49 with rank C (40.2%) (Table 1). The incidence of recurrence was significantly higher in rank C than in rank A or B (*p* = 0.0014). Based on this result, the following comparison was performed in two groups: rank A + B vs. rank C.

**Fig. 1:**
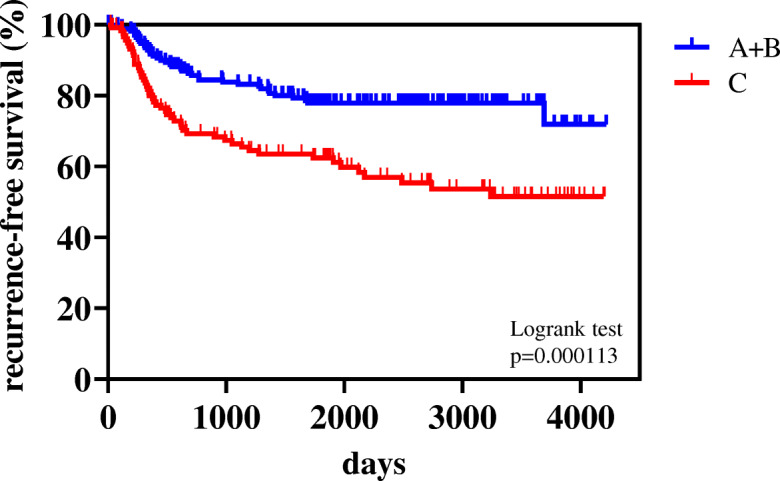
Kaplan-Meier curves for recurrence-free survival (RFS) of pre-operative AICS (lung) rank A + B and rank C

**Fig. 2:**
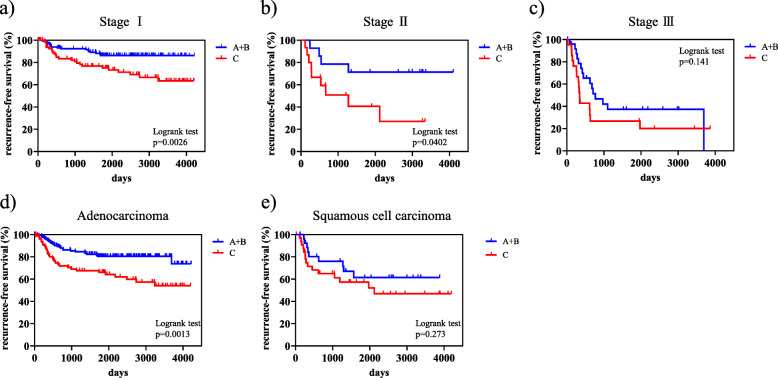
Kaplan-Meier curves for recurrence-free survival (RFS) of pre-operative AICS (lung) rank A + B and rank C according to the p-stage and histological type. **a** p-stage I, **b** p-stage II, **c** p-stage III, **d** Adenocarcinoma, **e** Squamous cell carcinoma

The 5-year RFS rate in all patients was 71.7%. RFS curves in ranks A + B and C were shown in Fig. 1. Five-year-RFS rates were 79.9% in rank A + B and 62.5% in rank C, and were significantly different (*p* = 0.000113). Figure 2 shows the RFS curves of these two groups according to the p-stage and histological type. A significant difference was observed in p-stage I (Fig. 1-a, *p* = 0.0026) and p-stage II (Fig. 2-b, *p* = 0.0402), but not in p-stage III (Fig. 2-c, *p* = 0.141). Regarding the histological type, only adenocarcinoma showed a significant difference in RFS (Fig. 2-d and 2-e, *p* = 0.0013). According to the univariate analysis of RFS in the present study, significant variables were the smoking status (non vs. current/ex), surgical mode (limited vs. standard), stage (I vs. II-IV), histological type (non-squamous cell carcinoma, non-Sq vs. squamous cell carcinoma, Sq), treatment method (surgery alone vs. surgery + adjuvant therapy), and preoperative AICS (lung) rank (rank A + B vs. rank C) (Table 2). Table 3 shows a multivariate analysis of RFS. Of the six variables tested, the p-stage, AICS (lung) rank, and treatment method were identified as significantly independent factors affecting RFS.

**Table 2:**
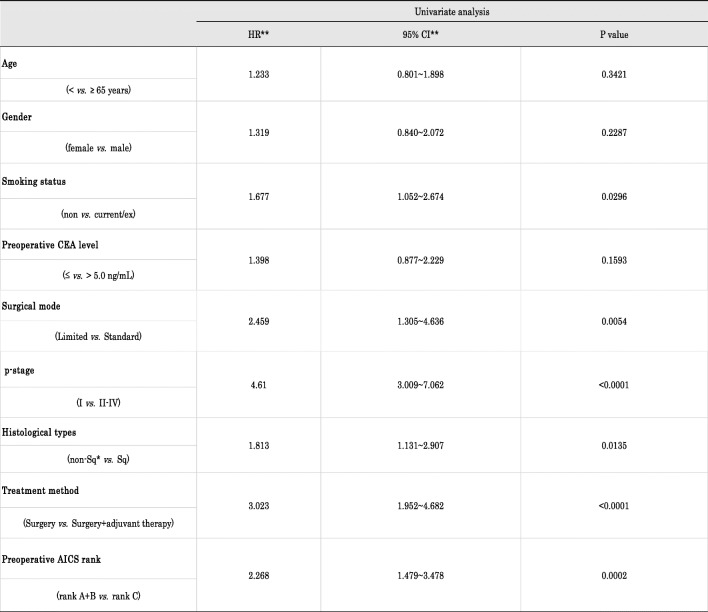
Univariate analysis of clinicopathological factors and recurrence-free survival (RFS)

* non-Sq, adenocarcinoma + others

** HR, hazard ratio; CI, confidence interval

**Table 3:**
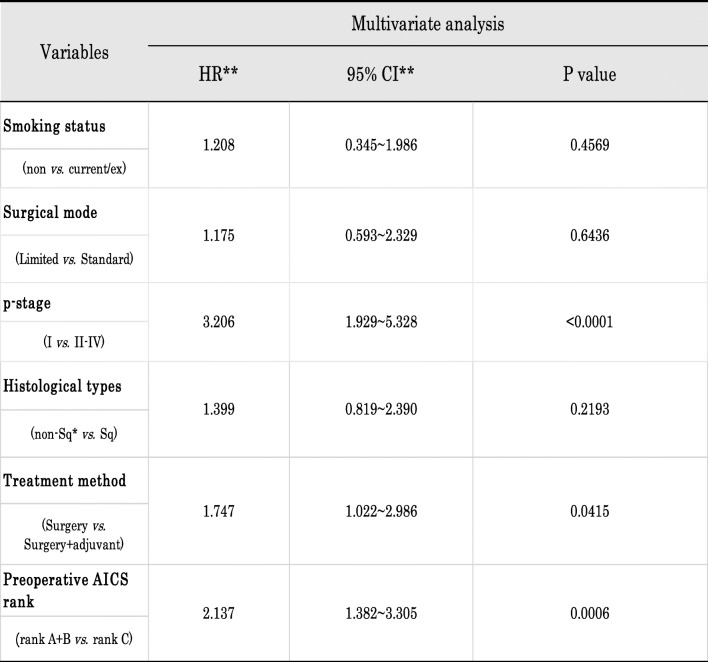
Multivariate analysis of clinicopathological factors and recurrence-free survival (RFS)

* non-Sq, adenocarcinoma + others

** HR, hazard ratio; CI, confidence interval

**Fig. 3:**
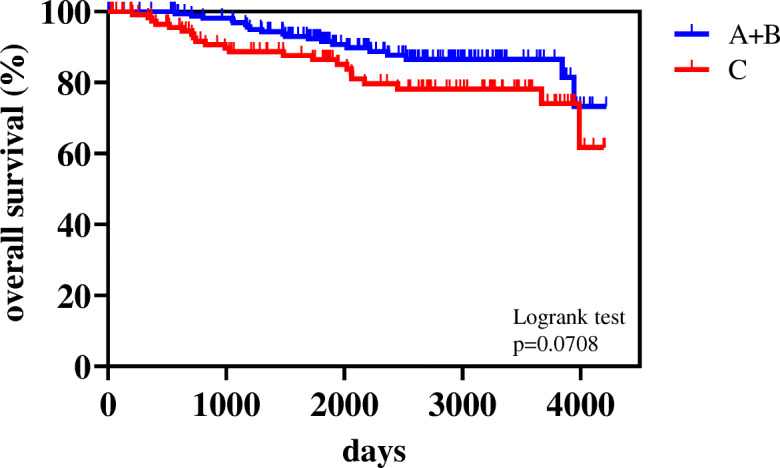
Kaplan-Meier curves for overall survival (OS) of pre-operative AICS (lung) rank A + B and rank C

Forty-two (14.1%) patients died. Death was not associated with the AICS rank (Table 1). The 5-year OS rates were 89.5% in all patients, 91.5% in rank A + B, and 86.5% in rank C. A slight difference was observed in OS between rank A + B and rank C (Fig. 3, *p* = 0.0708).

### Each AICS (lung) rank and the initial recurrence site

Since a marginal difference was found in surgical mode among each rank (Table 1), the postoperative recurrence pattern was carefully examined. Table 4 shows the relationship between the AICS (lung) rank and initial recurrence site in this cohort. No significant differences were observed in the recurrence site between rank A + B and rank C.

**Table 4:**
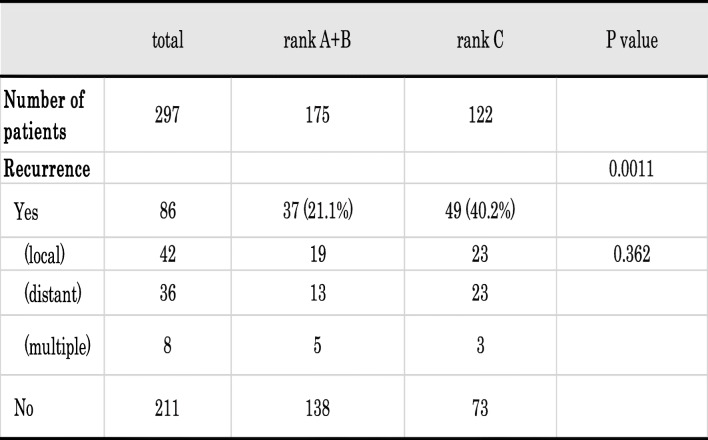
Recurrence pattern according to the AICS (lung) rank

## Discussion

AminoIndex™ technology was originally developed to screen various types of diseases, such as cancer [1, 7, 15, 16], hepatic or renal failure [17], cardiovascular and metabolic disorders [18–20], and others [21]. The hypothesis for its development was based on the highly sensitive detection of minute changes in metabolic profiles with a focus on PFAA, even if the disease was in the early stages. The AICS™ test is currently performed to detect 7 types of cancers, and the AICS (lung) test for lung cancer is widely used in health and cancer screening in Japan. Several promising studies on similar or the same concepts have been reported abroad [5, 21–25]. According to our previous findings [1, 8, 9], this test may be an optional tool for the early detection of lung cancer, regardless of the tumor stage or progression, in contrast to other tumor markers, such as serum CEA and CYFRA 21-1. However, since the mechanisms responsible for AICS (lung) abnormalities, namely, increases in AICS (lung) values with the occurrence or progression of lung cancer, remain unclear, the usefulness of this test has not yet been examined in detail in clinical practice. For example, clinicopathological differences between patients with and without this AICS (lung) abnormalities remain unclear.

In the present study, we examined the clinicopathological and prognostic significance of AICS (lung) abnormalities in 297 patients with NSCLC, who underwent curative surgery in our two institutes. The preoperative AICS (lung) status in patients with resectable NSCLC was observed on a relatively large scale: 93 with rank A (31.3%), 82 with rank B (27.7%), and 122 with rank C (41.1%). This distribution was similar to that in our previous study [10]. No relationships were observed between the preoperative AICS (lung) rank and clinicopathological factors, except for patient age and surgical mode. Consistent with previous findings [1, 2, 8, 9], the present results confirmed that AICS (lung) abnormalities was not related to the tumor stage at the start of surgical treatment. Interestingly, preoperative AICS (lung) abnormalities themselves had a significant effect on the frequency of postoperative recurrence among NSCLC patients undergoing curative surgery: patients diagnosed with AICS (lung) rank C in preoperative blood samples showed a significantly higher incidence of tumor recurrence even after complete resection than those diagnosed with the two other ranks. Although a significant impact on OS was not observed, the AICS (lung) rank was associated with the grade of tumor malignancy. This result was more apparent in patients with early-stage disease (p-stage I and II), particularly those with adenocarcinoma. This is the first study to investigate the clinical relationship between the preoperative amino acid profile status and tumor recurrence.

In the multivariate analysis, three variables (p-stage, treatment method, and AICS rank) were identified as significant high-risk predictors of tumor recurrence in the present cohort. As described previously [26], the p-stage had the greatest impact on recurrence after curative surgery. It currently remains unclear why the treatment method was regarded as a high-risk predictor; however, a bias was speculated because patients treated with surgery plus adjuvant therapy were intentionally selected as having a high-grade tumor malignancy and potentially unfavorable prognosis in each stage. In contrast, the AICS (lung) rank was firstly suspected to be a confounder associated with the surgical mode. In fact, limited surgery was more selected for rank A + B. Considering that the predictive power of the AICS (lung) rank was mainly limited to a subpopulation of patients with stage I and/or adenocarcinoma, it was also suspected that most patients with adenocarcinoma subtype indicating a predominant ground glass opacity (GGO) on preoperative CT scan image and/or a predominantly lepidic pattern in histology were included in rank A + B. However, such a prognostic good adenocarcinoma subtype was distributed regardless AICS (lung) rank (Supplementary Table 1). Therefore, the importance of preoperative AICS (lung) abnormalities was clearly determined in a multivariate analysis.

We recently reported that most patients preoperatively diagnosed with rank C potentially exhibited a reduction in the AICS (lung) rank if they were cured after surgery [10]. Furthermore, similar findings were reported for other types of cancers [27, 28]. In contrast, some NSCLC patients without AICS (lung) abnormalities in the preoperative test, namely, those diagnosed with rank A or B, also showed tumor recurrence, even after curative resection. These findings demonstrated that the condition of AICS (lung) abnormalities themselves may not only reflect the tumor-bearing status in patients, but may also be strongly associated with some biological grade of malignant characteristics associated with tumor proliferation, invasiveness, and/or metastasis [4, 10, 27, 28]. However, no significant differences were observed in recurrence patterns in the present cohort, suggesting that AICS (lung) abnormalities are not associated with the metastatic route.

In contrast to the results obtained for RFS, AICS (lung) abnormalities only slightly affected OS. We speculated that this discrepancy was mainly due to some therapeutic effects after recurrence. However, typical biomarkers for adenocarcinoma, such as EGFR mutations and ALK abnormalities, were not adequately examined because of the old times of some enrolled patients. The potential relationship between AICS (lung) abnormalities and these therapeutic biomarkers needs to be examined in future studies.

There were some limitations that need to be addressed. Although the number of enrolled patients was relatively large, the predictive value of AICS (lung) abnormalities for tumor recurrence was not satisfactory due to the retrospective nature of the analysis. Since its effect on tumor recurrence was limited in patients with early-stage disease and adenocarcinoma, the number of patients was too small to assess its significance. Moreover, the biological mechanisms and condition of AICS (lung) abnormalities in human cancer currently remain unknown. Several amino acids may exert promoting or inhibitory effects on the proliferation of cancer cells [4]. Alanine is regarded as an important key amino acid in apoptosis as well as the proliferation of cancer cells in vitro [4]. Moreover, according to recent findings on novel tissue free amino acid (TFAA) profile analyses [24, 29], the TFAA profile status was strongly associated with malignant characteristics as well as carcinogenesis in cancer patients, and PFAA profiles have been suggested to reflect the status of cancer tissues. Nevertheless, the present study was the first to show that minute changes and/or alterations in PFAA profiles are strongly associated with the biological grade of malignant characteristics. To clarify the predictive significance of AICS (lung) abnormalities on tumor recurrence in NSCLC patients undergoing surgical treatment, not only further validation in other cancers, but also basic research of the underlying mechanisms is warranted.

## Conclusions

The present study showed that preoperative AICS (lung) rank C was a high-risk predictor of postoperative recurrence in NSCLC patients undergoing curative resection. Although AICS was originally regarded as a screening modality for the risk of cancer, the AICS abnormalities may have a biological role in increasing the grade of malignant characteristics in cancer patients.

## Supplementary Information

**Additional file 1.**

## Data Availability

All data generated or analyzed during this study are included in this published article, and its supplementary information file.

## Abbreviations

AICS: AminoIndex Cancer Screening
AICS (lung) test: AminoIndex™ Cancer Screening test
ALK: Anaplastic lymphoma kinase
CEA: Carcinoembryonic antigen
CT: Computed tomography
CYFRA21-1: Cytokeratin 19 fragment
EGFR: Epidermal growth factor receptor.
FDG-PET: Fluoro-2-deoxyglucose positron emission tomography
GEM: Gemcitabine
HR: Hazard ratio
non-Sq: Non-squamous cell carcinoma
NSCLC: Non-small cell lung cancer
OS: Overall survival
PFAA: Plasma-free amino acid
RFS: Recurrence free survival
Sq: Squamous cell carcinoma
TFAA: Tissue free amino acid
TNM: Tumor node metastasis
UFT: Tegafur-Uracil
VNR: Vinorelbine
