# Supplementary material for: Preoperative AminoIndex Cancer Screening (AICS) abnormalities predict postoperative recurrence in patients undergoing curative resection for non-small cell lung cancer
Source: BMC Cancer. 2020 Nov 12;20:1100. doi: 10.1186/s12885-020-07575-w (PMC7659101; doi:10.1186/s12885-020-07575-w)
Supplement: Supplementary file 1 — Additional file 1. [file 12885_2020_7575_MOESM1_ESM.pptx]

## Slide 1
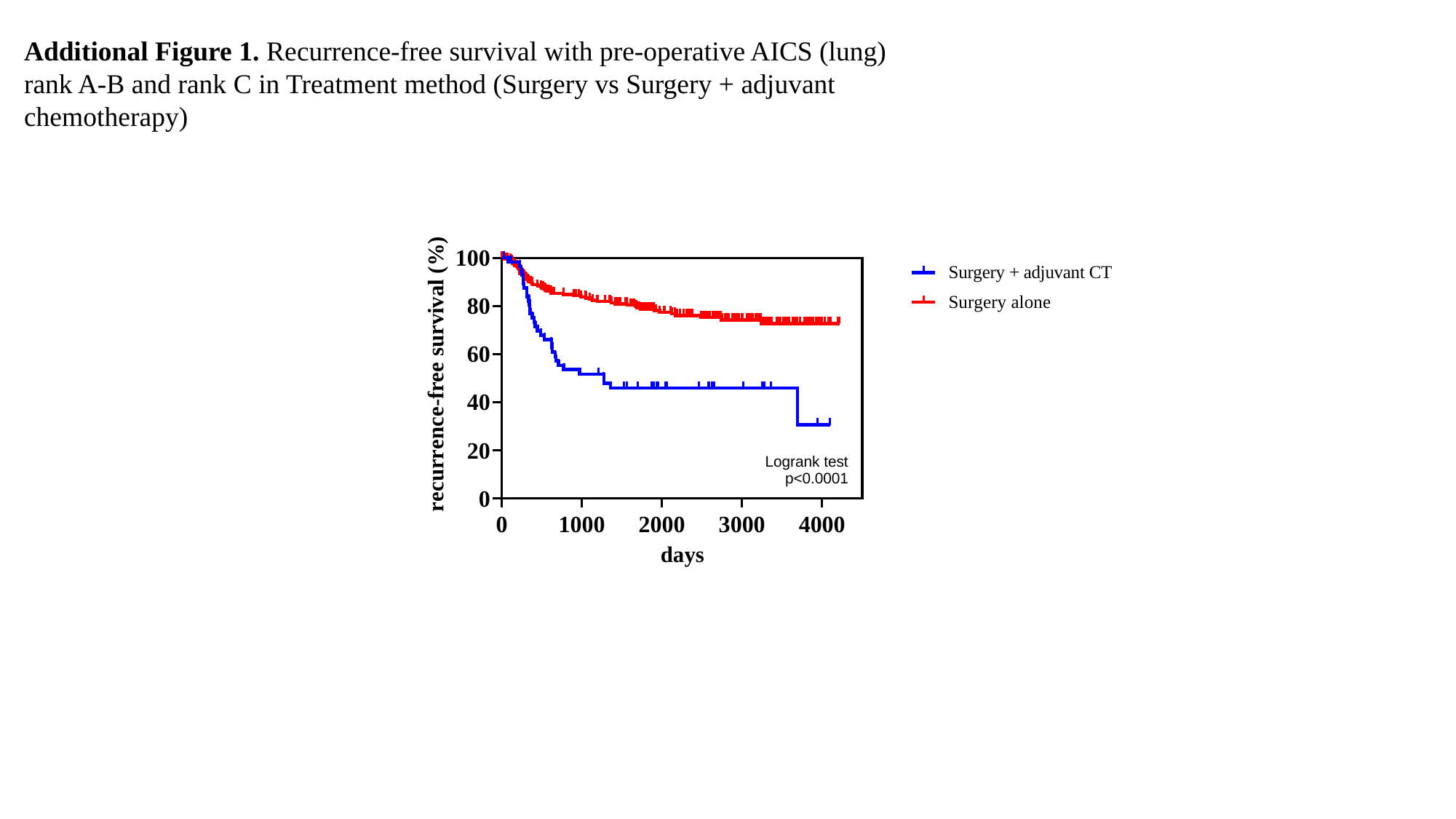

Additional Figure 1. Recurrence-free survival with pre-operative AICS (lung) rank A-B and rank C in Treatment method (Surgery vs Surgery + adjuvant chemotherapy)
| Logrank test p<0.0001 |
| --- |

## Slide 2
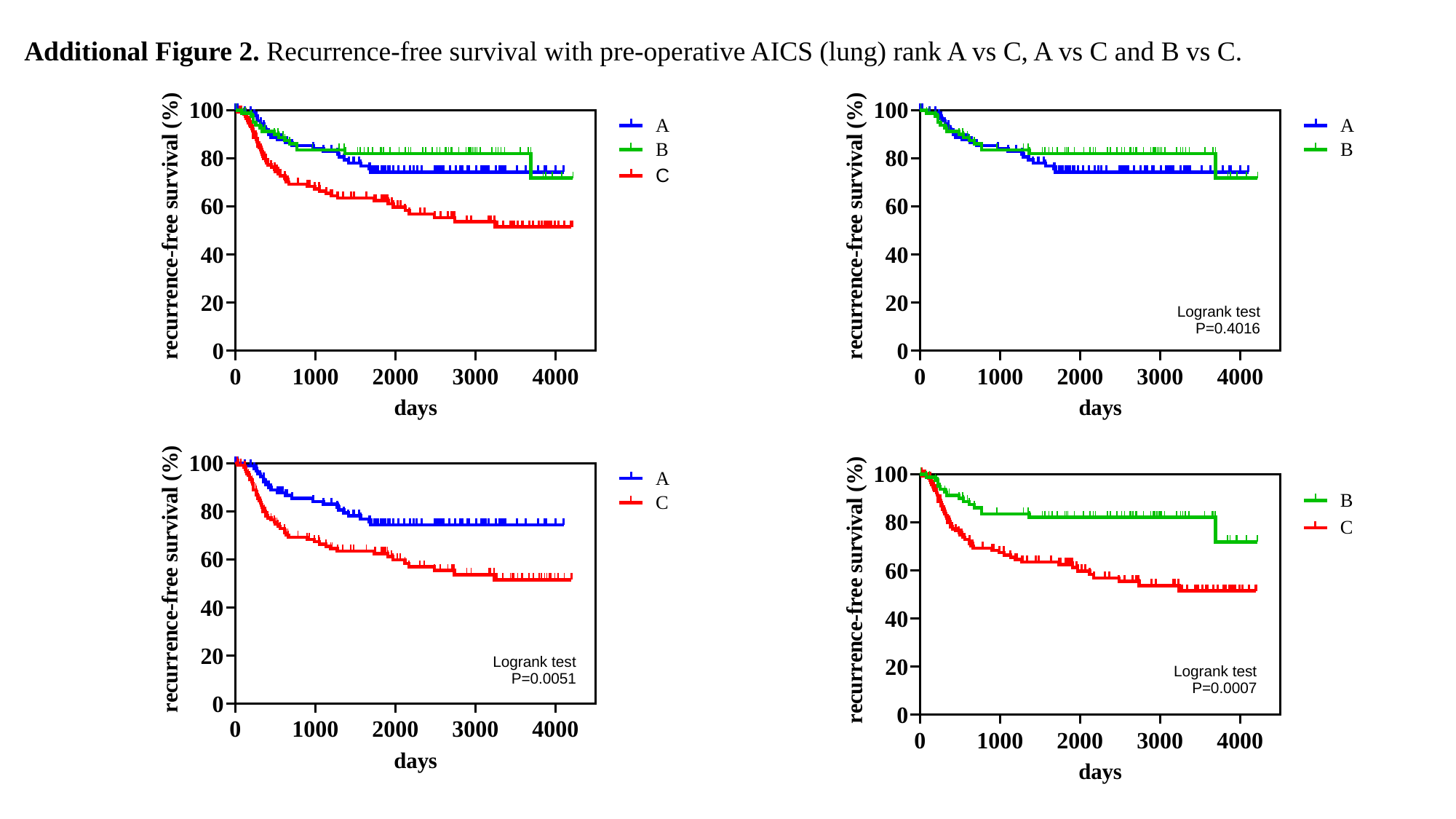

Additional Figure 2. Recurrence-free survival with pre-operative AICS (lung) rank A vs C, A vs C and B vs C.
| Logrank test P=0.4016 |
| --- |
| Logrank test P=0.0051 |
| --- |
| Logrank test P=0.0007 |
| --- |

## Slide 3
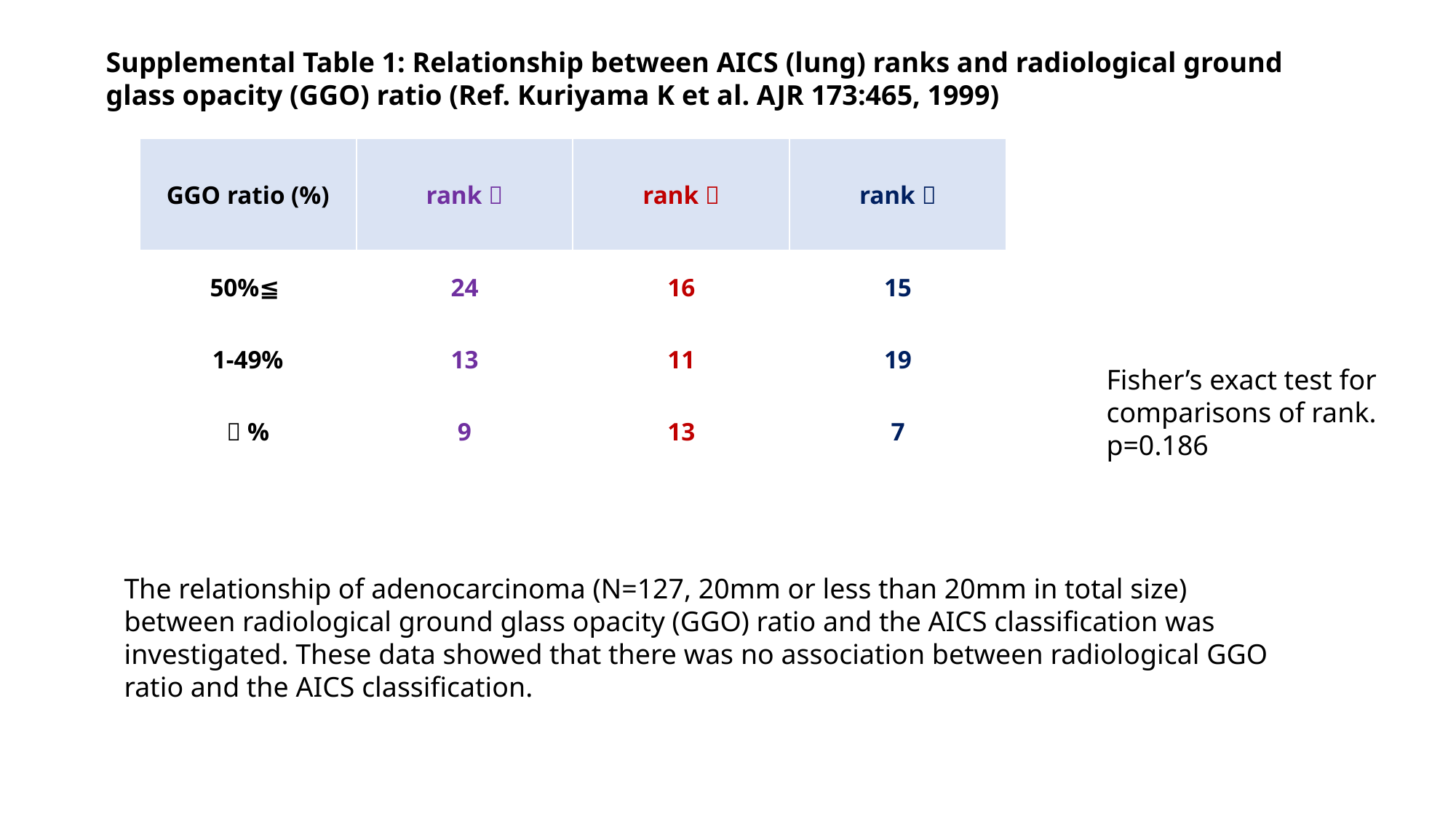

Supplemental Table 1: Relationship between AICS (lung) ranks and radiological ground glass opacity (GGO) ratio (Ref. Kuriyama K et al. AJR 173:465, 1999)
| GGO ratio (%) | rankＡ | rankＢ | rankＣ |
| --- | --- | --- | --- |
| 50%≦ | 24 | 16 | 15 |
| 1-49% | 13 | 11 | 19 |
| ０% | 9 | 13 | 7 |
Fisher’s exact test for comparisons of rank.
p=0.186
The relationship of adenocarcinoma (N=127, 20mm or less than 20mm in total size) between radiological ground glass opacity (GGO) ratio and the AICS classification was investigated. These data showed that there was no association between radiological GGO ratio and the AICS classification.
